# First-in-human phase I study of CLL-1 CAR-T cells in adults with relapsed/refractory acute myeloid leukemia

**DOI:** 10.1186/s13045-022-01308-1

**Published:** 2022-07-07

**Authors:** Xin Jin, Meng Zhang, Rui Sun, Hairong Lyu, Xia Xiao, Xiaomei Zhang, Fan Li, Danni Xie, Xia Xiong, Jiaxi Wang, Wenyi Lu, Hongkai Zhang, Mingfeng Zhao

**Affiliations:** 1 Tianjin First Central Hospital, The First Affiliated Central Hospital of Nankai University, The First Central Clinical College of Tianjin Medical University, Tianjin, 300192 China; 2 School of Medicine, Nankai University, Tianjin, 300071 China; 3 State Key Laboratory of Medicinal Chemical Biology and College of Life Science, Nankai University, Tianjin, 300350 China

**Keywords:** Chimeric antigen receptor, Acute myeloid leukemia, C-type lectin-like molecule 1

## Abstract

Relapsed or refractory (R/R) acute myeloid leukemia (AML) has a poor prognosis. In this study, we evaluated chimeric antigen receptor (CAR) T cell therapy targeting CLL-1 in adults with R/R AML patients. Patients received conditioning chemotherapy with cyclophosphamide (500 mg/m^2^) and fludarabine (30 mg/m^2^) for 3 days and an infusion of a dose of 1–2 × 10^6^ CAR-T cells/kg. The incidence of dose-limiting toxicity was the primary endpoint. Ten patients were treated, and all developed cytokine release syndrome (CRS); 4 cases were low-grade, while the remaining 6 were considered high-grade CRS. No patient developed CAR-T cell-related encephalopathy syndrome (CRES). Severe pancytopenia occurred in all patients. Two patients died of severe infection due to chronic agranulocytosis. The complete response (CR)/CR with incomplete hematologic recovery (CRi) rate was 70% (*n* = 7/10). The median follow-up time was 173 days (15–488), and 6 patients were alive at the end of the last follow-up. CAR-T cells showed peak expansion within 2 weeks. Notably, CLL-1 is also highly expressed in normal granulocytes, so bridging hematopoietic stem cell transplantation (HSCT) may be a viable strategy to rescue long-term agranulocytosis due to off-target toxicity. In conclusion, this study is the first to demonstrate the positive efficacy and tolerable safety of CLL-1 CAR-T cell therapy in adult R/R AML.

**Supplementary Information:**

The online version contains supplementary material available at 10.1186/s13045-022-01308-1.

To the Editor,

R/R AML patients have poor long-term survival [1, 2]. T cells expressing CAR have been recognized as a promising approach for hematological malignancies [3], but the effects of CAR-T cell therapy in R/R AML are limited and need to be improved [4, 5]. Human C-type lectin-like molecule 1 (CLL-1) is expressed on malignant cells in more than 90% of AML patients, but is underexpressed in normal hematopoietic stem cells [6]. Previous studies have shown that targeting CLL-1 can treat AML in preclinical studies [7–9], and 3 of 4 children with refractory AML who received CLL-1 CAR-T cells achieved CR [10]. Here, we report the first clinical trial of CLL-1 CAR-T cells in adult patients with R/R AML, recruiting 10 patients with positive efficacy and tolerable safety after cell infusion.

## Patient characteristics and CLL-1 CAR-T cell

Ten patients with relapsed/refractory AML were enrolled. The median age of all patients was 43.5 years (range 18–73), 8 of 10 patients had relapse, and 5 of them relapsed after transplantation. Three had MDS-to-AML transformation. The median of previous treatment lines was 5 (range 2–10), and all patients were resistant to the most recent chemotherapy before receiving CLL-1 CAR-T cell therapy (Table 1). The median of positive expression rate of CLL-1 in tumor cells of all patients was 85.2% (range 50.2%-97.6%). The median CAR-T cell infection efficiency was 50.53% (range 23.98%-73.14%). The median dose of infused CAR-T cells was 1.5 × 10^6^/kg (range 1 × 10^6^–2 × 10^6^) (Additional file 1: Table S1).

**Table 1:** Characteristics of patients before CAR-T cell treatment

ID	Age/Sex	FAB subtype	Fusion gene	Gene mutation	Karyotype	History of MDS/MPN	Prior lines of treatment	Previous HSCT	Extramedullary invasion	Pre-infusion disease burden (%)	Post-infusion disease burden (%)/efficacy evaluation	Bridged HSCT	CLL-1 Positivity (%)
1	53/F	M5	Negative	KRAS、TET2、ETV6	46, XX, -7, + mar	Yes	6	No	Yes	16.43	71.78/NR	Yes	50.2
2	44/M	M2	N/A	NPM1、DNMT3A、IDH1、RUNX1、NRAS	N/A	No	8	No	No	13.47	35.42/NR	Yes	90.3
3	73/M	M5	Negative	RUNX1、CEBPA、TET2、ASXL1、NRAS	46, XY del (7), (q22q34)	Yes	2	No	No	18.49	0.23/CRi	No	82.8
4	29/M	M5	Negative	CEBPA、FLT3、TET2	N/A	No	2	No	Yes	14.50	0/CRi	Yes	92.3
5	47/F	M5	Negative	Negative	Normal	No	8	Yes	No	83.55	86.51/NR	No	89.6
6	49/F	M5	MLL-AF9	ZRSR2	N/A	No	2	No	No	28.36	0/CR	No	97.6
7	43/F	M2	Negative	Negative	N/A	No	10	Yes	No	7.12	0/CRi	No	82.2
8	39/F	M5	Negative	RUNX1, U2AF1	46, XX, + 1, der(1;7)	Yes	4	Yes	No	10.24	2.11/CRi	Yes	66.9
9	18/F	M2	Negative	RUNX1、FLT3	Normal	No	6	Yes	No	3.09	0/CRi	Yes	80.6
10	29/M	M5	Negative	Negative	Normal	No	3	Yes	No	22.80	3.02/CRi	Yes	87.6

*ID* identification number, *F* female, *M* male, *FAB* French–American–British, *N/A* not available, *MDS/MPN* myelodysplastic syndrome/myeloproliferative neoplasm, *HSCT* hematopoietic stem cell transplantation, *CR* complete response, *CRi* CR with incomplete blood count recovery

## Safety

**Fig. 1:**
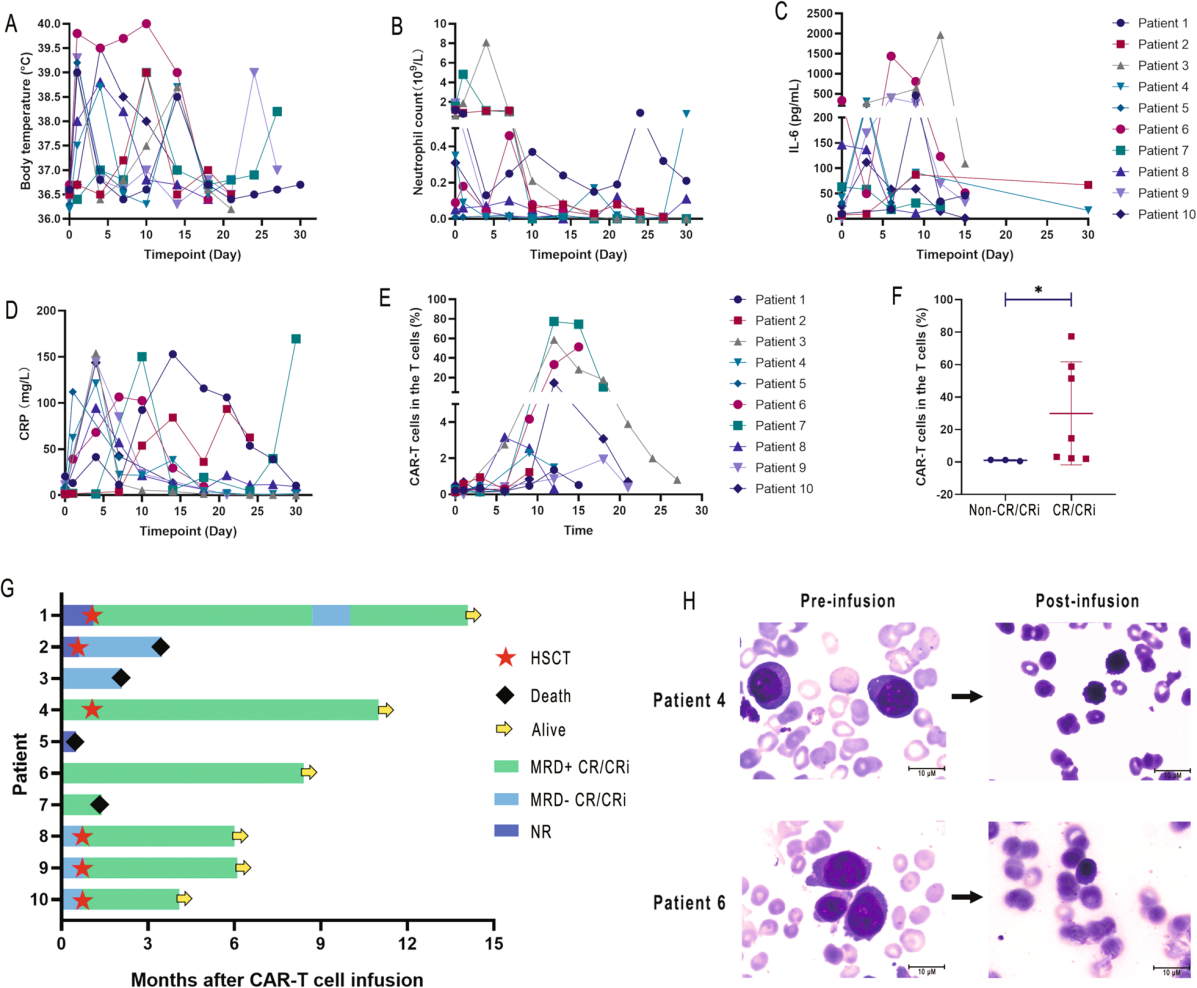
Kinetics of peripheral blood biomarkers and clinical outcome after CLL-1 CAR-T cell infusion. (**A**, **B**) Changes in patient body temperature and peripheral blood neutrophil numbers after CAR-T cell infusion, respectively. (**C**, **D**) Peripheral blood serum levels of IL-6, C-reactive protein (CRP) and ferritin before and after CAR-T cell infusion. (**E**) The ratio of CAR-T cells (CAR-T cells did not specifically distinguish between CD4 and CD8) to T cells in peripheral blood at various time periods. (**F**) Comparison of the peak values of CAR-T cells (CAR-T cells did not specifically distinguish between CD4 and CD8) in complete response (CR)/CRi and nonresponse (NR) patients. (**G**) Duration of response and survival after infusion of CLL-1 CAR-T cells. (**H**) Bone marrow smears of patient 4 and patient 6 before and after infusion. The data are expressed as the mean ± standard deviation (*p < 0.05)

Most patients developed fever during the infusion, which we consider to be an infusion-related reaction not related to CRS (Fig. 1A). The patient's fever almost always occurred in the range of 4–14 days after the infusion, which is correlated with the period of neutropenia (Fig. 1B). All patients developed CRS (Fig. 1C, D; Additional file 1: Fig. S1 A–C; Additional file 1: Table S1). CRS was controlled after 6/10 patients and 3/10 patients had received corticosteroids and tocilizumab, respectively. None of the 10 patients developed CRES. However, all patients had severe pancytopenia, 9/10 had grade 3/4 agranulocytosis, 7/10 had grade 3/4 anemia, and 7/10 had grade 3/4 thrombocytopenia (Additional file 1: Tables S2, S3). Patient 2 underwent salvage hematopoietic stem cell transplantation (HSCT) after achieving partial response (PR) with infusion and died of disease progression 2 months later. Patients 3 and 7 died of severe infection due to chronic agranulocytosis despite achieving CRi after therapy. Patient 5 died due to a nonresponse (NR) to treatment and rapid disease progression. Among the 6 patients who received bridging haploidentical transplantation after infusion (Fig. 1G). Granulocytes, erythroid and platelets all engrafted normally, and no serious infection occurred. Therefore, while severe agranulocytosis occurs after infusion, bridging transplantation may reverse this toxicity.

## Efficacy

7/10 patients achieved CR/CRi (Fig. 1G, H). The median follow-up time was 173 days (15–488), and 6 patients were alive at the end of the last follow-up (Table S1). Patient 1 had no response (possibly related to lower CLL-1 expression on her tumor cells) after infusion followed by salvage HSCT and achieved CR. However, she was found to have minimal residual disease (MRD) 9 months later and became MRD negative after azacytidine and venetoclax treatment. Patient 6 did not undergo other treatments after infusion, and the patient was in continuous CR during the follow-up. Six patients underwent HSCT at a median of 20 days after infusion (range: 18–34), and four (50%) were still CR at the last follow-up.

## Biomarker analysis

CLL-1 CAR-T cell expansion was assessed by flow cytometry. The median CAR-T cell expansion peaked at day 12 after reinfusion (range 6–18 days) (Fig. 1E). Comparing peak CAR-T cell expansion in CR/CRi and non-CR/CRi patients, the CR/CRi patients had significantly higher proportions of CAR-T cells (Fig. 1F). Cytokine levels were increased to varying degrees after infusion (Fig. 1C, D; Fig. S1 A-C). However, in non-CR/CRi patients, the detection values ​​of cytokines and CAR-T cells were relatively low.

In conclusion, CLL-1 may be a potential therapeutic target for AML. Although severe agranulocytosis may occur, CLL-1 CAR-T cell can provide R/R AML patients with the chance to achieve CR/CRi before transplantation, which may reduce the risk of relapse and prolong patient survival. Our study found that granulocytes are difficult to recover after infusion, which is inconsistent with a previous study in children [10], which may be due to the repopulating potential of hematopoietic stem cells in childhood AML patients.

## Supplementary Information

**Additional file 1.** Study methods and additional patient information.

## Data Availability

All data generated or analyzed during this study are included in this published article or its supplementary information files. The raw datasets are available from the corresponding authors on reasonable request.

## Abbreviations

R/R: Relapsed or refractory
AML: Acute myeloid leukemia
CAR: Chimeric antigen receptor
CRS: Cytokine release syndrome
CRES: Chimeric antigen receptor T cell-related encephalopathy syndrome
HSCT: Hematopoietic stem cell transplantation
CR: Complete response
CRi: CR with incomplete blood count recovery
CLL-1: C-type lectin-like molecule 1 (CLL-1)
MRD: Minimal residual disease
PR: Partial response

## References

[1] Rollig C (2011). Long-term prognosis of acute myeloid leukemia according to the new genetic risk classification of the European LeukemiaNet recommendations: evaluation of the proposed reporting system. J Clin Oncol.

[2] Rubnitz JE, Kaspers GJL (2021). How I treat pediatric acute myeloid leukemia. Blood.

[3] Young RM, Engel NW, Uslu U, Wellhausen N, June CH (2022). Next-generation CAR T-cell therapies. Cancer Discov.

[4] Mardiana S, Gill S (2020). CAR T cells for acute Myeloid Leukemia: state of the art and future directions. Front Oncol.

[5] Marvin-Peek J, Savani BN, Olalekan OO, Dholaria B (2022). Challenges and advances in chimeric antigen receptor therapy for acute Myeloid Leukemia. Cancers (Basel).

[6] Morsink LM, Walter RB, Ossenkoppele GJ (2019). Prognostic and therapeutic role of CLEC12A in acute myeloid leukemia. Blood Rev.

[7] Wang J (2018). CAR-T cells targeting CLL-1 as an approach to treat acute myeloid leukemia. J Hematol Oncol.

[8] Tashiro H (2017). Treatment of acute myeloid leukemia with T cells expressing chimeric antigen receptors directed to C-type lectin-like molecule 1. Mol Ther.

[9] Laborda E (2017). Development of A chimeric antigen receptor targeting C-type lectin-like molecule-1 for human acute myeloid leukemia. Int J Mol Sci.

[10] Zhang H (2021). Anti-CLL1 chimeric antigen receptor T-cell therapy in children with relapsed/refractory acute myeloid leukemia. Clin Cancer Res.

