# Supplementary material for: First-in-human phase I study of CLL-1 CAR-T cells in adults with relapsed/refractory acute myeloid leukemia
Source: J Hematol Oncol. 2022 Jul 7;15:88. doi: 10.1186/s13045-022-01308-1 (PMC9264641; doi:10.1186/s13045-022-01308-1)
Supplement: Supplementary file 1 — Additional file 1. Study methods and additional patient information. [file 13045_2022_1308_MOESM1_ESM.docx]

Supplementary Materials

**Materials**

Patients and Study Design

This study was an observational study that included a total of 10 patients with relapsed/refractory AML (ChiCTR2000041054). The main inclusion criteria for eligible patients were as follows: (1) patients were diagnosed with acute myeloid leukemia who were refractory to two rounds of induction chemotherapy or were in relapse after complete remission and considered to be R/R AML; (2) the tumor cells expressed CLL-1 as identified by flow cytometry; (3) single-cell collection could be used to obtain T cells for the preparation of CAR-T cells; (4) there was no active bacterial, fungal, or viral infection; and (5) the patient was able to sign an informed consent form. The main exclusion criteria were a history of malignant tumor and severe renal, hepatic and cardiac dysfunction.

CAR vector construction

The single-chain variable fragment (scFv) targeting CLL-1 originated from the M26 clone (patent WO2017091615A). The CAR vectors contained the scFv and human 4-1BB and CD3ζ signaling domains, which were subcloned into the pCDH-MND-MCS-T2A-Puro lentiviral plasmid vector. The CAR sequence was preceded by the RQR8 tag separated by a short T2A peptide for detection^1^.

Preparation of CAR-T Cells

Peripheral blood samples were obtained, and mononuclear cells were extracted with lymphocyte separation medium. The CD3+ T cells were enriched from the extracted mononuclear cells by immunomagnetic bead sorting. The obtained cells were cultured with T-cell-specific medium containing IL-2 and glutamine (day 0), and CD3/CD28 magnetic beads were added to activate T cells. CLL-1 CAR-T cells were prepared by inoculating lentivirus with different elements infected T cells on the 2nd to 3rd day of culture. The CLL-1 CAR-T cells target specific analysis was comprehensively conducted in our preclinical studies^2^.

Study Design

All patients received cyclophosphamide (500 mg/m^2^) and fludarabine (30 mg/m^2^) regimens for 3 days before CLL-1 CAR-T-cell therapy to enhance the proliferation of CAR-T cells in vivo. Bridging hematopoietic stem cell transplantation was performed in patients with consent. CRS patients received symptomatic treatment, and during the treatment period, if neutrophils were less than 0.5×10^9^/L, prophylactic antibiotics were given.

Clinical Response Assessment

For the first 3 months after CAR-T-cell therapy, the efficacy was assessed monthly by bone marrow morphology and flow cytometry, and peripheral blood and biochemical assessments were conducted at least twice a week. Evaluations were performed every 3 months after the 3rd month. The evaluation criteria were based on the NCCN guidelines for efficacy evaluation. CRS was graded according to the criteria of Lee et al^3^.

Cytokine analysis

The concentrations of serum inflammation markers IL-2, IL-4, IL-6, IL-10, TNF-α and IFN-γ were evaluated using the Luminex detection method according to the manufacturer's instructions. In short, add analysis buffer, sample serum and capture microsphere solution to the tube, and incubate in the dark for 2 hours. Add 1 ml of washing solution to the tube, centrifuge and discard the supernatant. Add the antibody in the kit and incubate for 1 hour in the dark (without washing). Add SA-PE and incubate in the dark. Add 1 ml of washing solution, centrifuge and discard the supernatant. Add 150μl washing solution, and analyze with flow cytometer (ACEA NovoCyte).

Statistical Analysis

The statistical analysis was mostly descriptive. For comparison of patient CAR-T-cell levels, a T test was used, and GraphPad Prism software was used for calculation. The significance level was set at 0.05. Data were analyzed as of May 15, 2022.

Figure S1


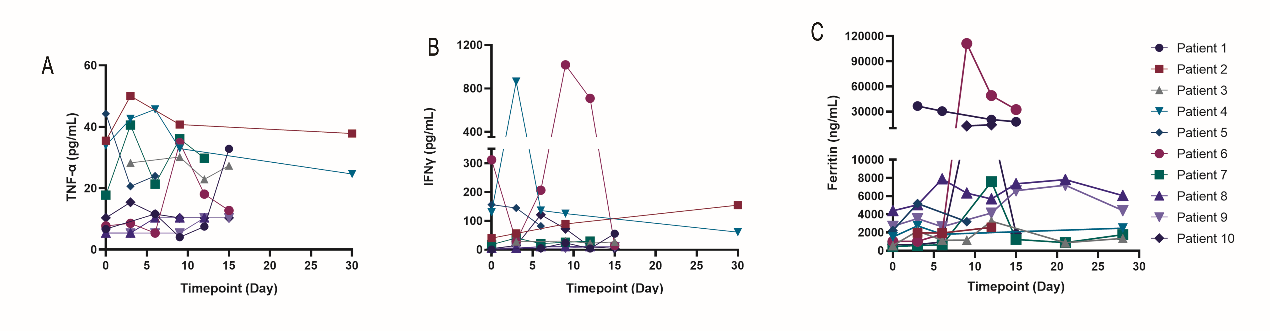


Fig S1 Serum biomarkers after CLL-1 CAR-T-cell infusion. Peripheral blood serum levels of TNF-α, IFNγ and ferritin before and after CAR-T-cell infusion.

**Table S1.** Patient outcome after CAR-T-cell treatment

| ID | Age/Sex | CRS/grade | CRES/grade | Interventions for CRS | % CAR transduction efficiency | CAR-T-cell dose (/kg) | Neutropenia/grade | Anemia (grade) | Thrombocytopenia (grade) | Response | Bridged transplantation | Status at last follow-up (days) |
| --- | --- | --- | --- | --- | --- | --- | --- | --- | --- | --- | --- | --- |
| 1 | 52/F | 2 | 0 | corticosteroids | 59.78 | 1×10^6^ | 4 | 3 | 4 | NR | Yes | CR (424) |
| 2 | 44/M | 2 | 0 | corticosteroids | 50.96 | 2×10^6^ | 3 | 3 | 4 | NR | Yes | Died of PD (126) |
| 3 | 73/M | 3 | 0 | corticosteroids, tocilizumab | 31.60 | 2×10^6^ | 2 | 3 | 4 | MRD+ CRi | No | Died of infection (63) |
| 4 | 29/M | 3 | 0 | corticosteroids, tocilizumab | 52.07 | 1.5×10^6^ | 4 | 3 | 2 | MRD- CRi | Yes | CR (265) |
| 5 | 47/F | 2 | 0 |  | 26.79 | 2×10^6^ | 4 | 3 | 4 | NR | No | Died of PD (15) |
| 6 | 49/F | 3 | 0 | corticosteroids | 40.51 | 1×10^6^ | 4 | 3 | 1 | MRD- CR | No | CR (187) |
| 7 | 43/F | 3 | 0 | corticosteroids | 50.09 | 1×10^6^ | 4 | 1 | 1 | MRD- CRi | No | Died of infection (41) |
| 8 | 39/F | 2 | 0 |  | 23.98 | 2×10^6^ | 4 | 3 | 3 | MRD+ CRi | Yes | CR (138) |
| 9 | 18/F | 3 | 0 | tocilizumab | 73.14 | 1.5×10^6^ | 4 | 1 | 3 | MRD- CRi | Yes | CR (142) |
| 10 | 29/M | 3 | 0 |  | 70.40 | 1×10^6^ | 4 | 1 | 3 | MRD+ CRi | Yes | CR (80) |

ID: identification number; F: female; M: male; CRS: cytokine release syndrome; CRES: CART-cell-related encephalopathy syndrome. CR: complete response; NR: nonresponse; MRD: minimal residual disease.

CRS was graded per a modified grading system proposed by Lee et al.

Individual symptoms of CRS were graded per CTCAE version 5.0.

**Table S2. Treatment-emergent** **adverse events**

| Event | Any grade n (%) | Grade 3 n (%) | Grade 4 n (%) |
| --- | --- | --- | --- |
| Any grade 3 or higher AE | 10 (100) | 0 | 10 (100) |
| Encephalopathy | 0 | 0 | 0 |
| Anemia | 10 (100) | 7 (70) | 0 |
| Neutropenia | 10 (100) | 1 (10) | 8 (80) |
| Hypoxia | 1 (10) | 0 | 0 |
| Thrombocytopenia | 10 (100) | 3 (30) | 4 (40) |
| Acute kidney injury | 0 | 0 | 0 |
| Increased aspartate aminotransferase level | 3 (30) | 0 | 0 |
| Cardiac failure | 1 (10) | 0 | 0 |
| Delirium | 0 | 0 | 0 |
| Fatigue | 3 (30) | 0 | 0 |
| Intracranial hemorrhage | 0 | 0 | 0 |
| Hypocalcemia | 4 (40) | 0 | 0 |
| Hyponatremia | 1 (10) | 0 | 0 |
| Hypophosphatemia | 6 (60) | 0 | 0 |
| Hypotension | 2 (20) | 0 | 0 |
| Metabolic acidosis  Oral herpes | 2 (20) | 0 | 0 |
| Pseudomonal sepsis | 1 (10) | 0 | 1 (10) |
| Restlessness | 0 | 0 | 0 |
| Tremor | 0 | 0 | 0 |
| Urinary tract infection | 0 | 0 | 0 |
| Edema | 2 (20) | 0 | 0 |

AE: adverse events

Table S3.

Blood routine and Blood biochemistry during CAR-T cell Therapy.

|  | **Infusion time** | **Pre-infusion** | **1** | **4** | **7** | **10** | **14** | **18** | **21** | **24** | **28** | **35** | **42** | **49** |
| --- | --- | --- | --- | --- | --- | --- | --- | --- | --- | --- | --- | --- | --- | --- |
| **Patient 1** | Lymphocyte count (X10^9^/L) | 1.22 | 1.35 | 1.58 | 2.66 | 1.32 | 1.55 | 2.17 | 2.88 | 3.06 | 0.03 | 0.03 | 0.033 | 0.27 |
|  | Leukocyte count (X10^9^/L) | 3.19 | 3.25 | 2.96 | 2.97 | 3.35 | 3.25 | 4.07 | 5 | 6.06 | 5.16 | 0.39 | 0.06 | 3 |
|  | Hemoglobin (g/L) | 66 | 55 | 70 | 77 | 54 | 57 | 66 | 67 | 52 | 68 | 84 | 81 | 78 |
|  | Platelet (X10^9^/L) | 14 | 7 | 27 | 7 | 13 | 15 | 20 | 13 | 16 | 13 | 109 | 40 | 56 |
|  |  |  |  |  |  |  |  |  |  |  |  |  |  |  |
|  | ALT（U/L） | 10.5 | 9.8 | 9.5 | 11.8 | 12.9 | 15.5 | 14.9 | 19.8 | 21.1 | 38.9 | 18.4 | 10.2 | 23.2 |
|  | AST（U/L） | 8.9 | 11.4 | 8.8 | 7.8 | 12.6 | 9.9 | 10.3 | 15.5 | 11.7 | 21.9 | 13 | 10.2 | 28.1 |
|  | Total bilirubin (umol/L) | 3.36 | 5.43 | 5.2 | 5.61 | 2.7 | 7.21 | 6.67 | 6.5 | 4.17 | 6.87 | 17.25 | 11.3 | 14.59 |
|  | Direct bilirubin (umol/L) | 1.5 | 2.23 | 2.46 | 2.86 | 1.67 | 3.64 | 3.67 | 2.92 | 2.08 | 3.58 | 5.38 | 5.52 | 5.37 |
|  | Creatinine (umol/L) | 42 | 46 | 40 | 47 | 49 | 46 | 44 | 53 | 58 | 55 | 41 | 39 | 37 |
|  | LDH（U/L） | 253.2 | 261.9 | 248.9 | 234.4 | 297.9 | 290.7 | 279.6 | 238.6 | 265.5 | 282.5 | 223.8 | 149.9 | 774.3 |
|  |  |  |  |  |  |  |  |  |  |  |  |  |  |  |
| **Patient 2** | Lymphocyte count (X10^9^/L) | 0.3 | 0.21 | 0.16 | 0.29 | 0.06 | 0.09 | 0.02 | 0.01 | 0 | 0.01 |  |  |  |
|  | Leukocyte count (X10^9^/L) | 1.59 | 1.18 | 1.35 | 1.51 | 0.21 | 0.24 | 0.06 | 0.1 | 0.04 | 0.02 |  |  |  |
|  | Hemoglobin (g/L) | 56 | 62 | 63 | 65 | 53 | 68 | 70 | 64 | 72 | 71 |  |  |  |
|  | Platelet (X10^9^/L) | 12 | 10 | 32 | 13 | 9 | 31 | 10 | 39 | 27 | 32 |  |  |  |
|  |  |  |  |  |  |  |  |  |  |  |  |  |  |  |
|  | ALT（U/L） | 51.6 | 15.3 |  | 10.8 | 16.5 | 14.6 | 21.8 | 25.3 | 48.2 |  |  |  |  |
|  | AST（U/L） | 20.7 | 8 |  | 8 | 11.3 | 29.1 | 36.5 | 48.5 | 26.9 |  |  |  |  |
|  | Total bilirubin (umol/L) | 6.39 | 5.94 |  | 3.36 | 2.31 | 2.51 | 4.2 | 9.8 | 13.1 |  |  |  |  |
|  | Direct bilirubin (umol/L) | 2.56 | 2.33 |  | 1.69 | 1.42 | 1.34 | 2.67 | 6.57 | 7.1 |  |  |  |  |
|  | Creatinine (umol/L) | 64 | 66 |  | 69 | 84 | 88 | 63 | 62 | 58 |  |  |  |  |
|  | LDH（U/L） | 214.9 | 170.1 |  | 187 | 310.8 | 604 | 917.4 | 618.4 | 433.4 |  |  |  |  |
|  |  |  |  |  |  |  |  |  |  |  |  |  |  |  |
| **Patient 3** | Lymphocyte count (X10^9^/L) | 0.15 | 0.07 | 0.44 | 0.24 | 0.07 | 0.77 | 0.12 |  | 0.21 |  | 0.09 |  |  |
|  | Leukocyte count (X10^9^/L) | 0.83 | 2.04 | 8.67 | 1.51 | 0.31 | 0.81 | 0.13 | 0.1 | 0.22 | 0.09 | 0.09 |  |  |
|  | Hemoglobin (g/L) | 76 | 79 | 84 | 75 | 68 | 77 | 64 | 67 | 93 | 72 | 70 |  |  |
|  | Platelet (X10^9^/L) | 4 | 12 | 27 | 14 | 5 | 3 | 21 | 17 | 14 | 15 | 22 |  |  |
|  |  |  |  |  |  |  |  |  |  |  |  |  |  |  |
|  | ALT（U/L） | 7.9 |  | 12.2 | 18.8 | 37.7 | 44.8 | 25.4 |  | 11.8 | 17.3 | 16.5 |  |  |
|  | AST（U/L） | 10.2 |  | 17.7 | 17 | 30 | 48.2 | 21.5 |  | 9.9 | 12.6 | 9.4 |  |  |
|  | Total bilirubin (umol/L) | 14.35 |  | 24.95 | 19.62 | 28.33 | 23.72 | 28.37 |  | 30.5 | 24.58 | 23.5 |  |  |
|  | Direct bilirubin (umol/L) | 5.62 |  | 12.25 | 8.32 | 12.75 | 11.68 | 10.68 |  | 10.8 | 10.24 | 9.99 |  |  |
|  | Creatinine (umol/L) | 75 |  | 83 | 89 | 68 |  |  |  | 48 | 51 | 52 |  |  |
|  | LDH（U/L） | 146.2 |  | 236.6 | 191.8 | 272.9 | 314.4 |  |  | 124.4 | 109.4 | 80.2 |  |  |
|  |  |  |  |  |  |  |  |  |  |  |  |  |  |  |
| **Patient 4** | Lymphocyte count (X10^9^/L) | 0.83 | 0.03 | 0.04 | 0.09 | 0.21 | 0.01 | 0.01 | 0 | 0 | 0.01 | 0.17 | 0.11 |  |
|  | Leukocyte count (X10^9^/L) | 1.19 | 0.12 | 0.06 | 0.1 | 0.21 | 0.02 | 0.18 | 0.02 | 0.01 | 0.06 | 5.83 | 5.3 |  |
|  | Hemoglobin (g/L) | 63 | 62 | 50 | 54 | 74 | 76 | 72 | 63 | 73 | 69 | 73 | 96 |  |
|  | Platelet (X10^9^/L) | 81 | 35 | 19 | 26 | 109 | 262 | 224 | 127 | 41 | 11 | 164 | 212 |  |
|  |  |  |  |  |  |  |  |  |  |  |  |  |  |  |
|  | ALT（U/L） | 12.3 | 17.2 | 20.6 | 11.7 |  | 11.6 | 36.9 | 27.4 | 23.2 | 15.3 | 10.7 | 10 |  |
|  | AST（U/L） | 10.9 | 17.9 | 19.2 | 10.4 |  | 13.9 | 45.7 | 23.3 | 17.4 | 14.7 | 17.5 | 12.4 |  |
|  | Total bilirubin (umol/L) | 19.8 | 36 | 30.81 | 23.8 |  | 23.7 | 17.8 | 27.1 | 23.5 | 27 | 16.61 | 18.1 |  |
|  | Direct bilirubin (umol/L) | 8.79 | 11.18 | 9.75 | 8.52 |  | 9.28 | 8 | 11.65 | 10.39 | 11.39 | 7 | 7.8 |  |
|  | Creatinine (umol/L) | 55 | 61 | 61 | 52 |  | 55 | 48 | 56 | 53 | 69 | 56 | 52 |  |
|  | LDH（U/L） | 181 | 232.2 | 296.7 | 254.5 |  | 230 | 242.4 | 282 | 259 | 317.5 | 497.6 | 321.8 |  |
|  |  |  |  |  |  |  |  |  |  |  |  |  |  |  |
| **Patient 5** | Lymphocyte count (X10^9^/L) | 0.16 | 0.03 | 0.04 | 0.09 |  |  |  |  |  |  |  |  |  |
|  | Leukocyte count (X10^9^/L) | 0.19 | 0.07 | 0.15 | 0.17 |  |  |  |  |  |  |  |  |  |
|  | Hemoglobin (g/L) | 68 | 62 | 63 | 83 |  |  |  |  |  |  |  |  |  |
|  | Platelet (X10^9^/L) | 2 | 2 | 1 | 1 |  |  |  |  |  |  |  |  |  |
|  |  |  |  |  |  |  |  |  |  |  |  |  |  |  |
|  | ALT（U/L） | 8.9 |  | 12 |  |  |  |  |  |  |  |  |  |  |
|  | AST（U/L） | 12.5 |  | 15.9 |  |  |  |  |  |  |  |  |  |  |
|  | Total bilirubin (umol/L) | 8.74 |  | 12.28 |  |  |  |  |  |  |  |  |  |  |
|  | Direct bilirubin (umol/L) | 3.65 |  | 5.03 |  |  |  |  |  |  |  |  |  |  |
|  | Creatinine (umol/L) | 46 |  | 41 | 41 |  |  |  |  |  |  |  |  |  |
|  | LDH（U/L） | 182.2 |  | 254.5 | 269.4 |  |  |  |  |  |  |  |  |  |
|  |  |  |  |  |  |  |  |  |  |  |  |  |  |  |
| **Patient 6** | Lymphocyte count (X10^9^/L) | 0.33 | 0 | 0.21 | 0.29 | 0.28 | 0.65 | 0.32 |  |  |  |  |  |  |
|  | Leukocyte count (X10^9^/L) | 0.43 | 0 | 0.27 | 0.93 | 0.52 | 0.9 | 0.42 |  |  |  |  |  |  |
|  | Hemoglobin (g/L) | 68 | 69 | 66 | 63 | 67 | 67 | 73 |  |  |  |  |  |  |
|  | Platelet (X10^9^/L) | 17 | 5 | 8 | 67 | 66 | 216 | 578 |  |  |  |  |  |  |
|  |  |  |  |  |  |  |  |  |  |  |  |  |  |  |
|  | ALT（U/L） | 8.1 |  | 38.13 | 8.9 | 17.3 | 183.8 | 147.2 |  |  |  |  |  |  |
|  | AST（U/L） | 16.2 |  | 16.4 | 17.3 | 137.9 | 677.3 | 223.9 |  |  |  |  |  |  |
|  | Total bilirubin (umol/L) | 10.19 |  | 9.37 | 9.15 | 9.02 | 10 | 12.48 |  |  |  |  |  |  |
|  | Direct bilirubin (umol/L) | 5.23 |  | 5.07 | 5.22 | 5.85 | 6.08 | 6.19 |  |  |  |  |  |  |
|  | Creatinine (umol/L) | 43 |  | 47 | 49 | 47 | 53 | 57 |  |  |  |  |  |  |
|  | LDH（U/L） | 139 |  | 175.8 | 324.6 | 2890 | 2460 | 1300 |  |  |  |  |  |  |
|  |  |  |  |  |  |  |  |  |  |  |  |  |  |  |
| **Patient 7** | Lymphocyte count (X10^9^/L) | 0.47 | 0.26 | 0.43 | 0.35 | 0.74 | 0.41 | 0.31 | 0.25 | 0.28 | 0.17 | 0.19 | 0.06 |  |
|  | Leukocyte count (X10^9^/L) | 2.92 | 5.93 | 2.68 | 2.39 | 1.16 | 0.43 | 0.3 | 0.25 | 0.28 | 0.33 | 0.19 | 0.07 |  |
|  | Hemoglobin (g/L) | 104 | 107 | 112 | 121 | 119 | 98 | 104 | 108 | 110 | 108 | 91 | 70 |  |
|  | Platelet (X10^9^/L) | 82 | 89 | 72 | 92 | 33 | 42 | 70 | 50 | 51 | 38 | 6 | 1 |  |
|  |  |  |  |  |  |  |  |  |  |  |  |  |  |  |
|  | ALT（U/L） | 140.8 | 191.2 | 180.7 |  | 249.8 | 87.2 | 65.6 |  | 36.4 | 39.7 | 20.7 | 11.1 |  |
|  | AST（U/L） | 248.5 | 138.3 | 118.7 |  | 288.8 | 33.3 | 30.1 |  | 15.6 | 22.3 | 13.7 | 10.4 |  |
|  | Total bilirubin (umol/L) | 16.3 | 13.42 | 17.44 |  | 32.16 | 18.7 | 29.51 |  | 17.7 | 24.12 | 17.3 | 25.3 |  |
|  | Direct bilirubin (umol/L) | 6.47 | 6.56 | 7.87 |  | 15.55 | 9.47 | 10.94 |  | 8.72 | 8.63 | 8.51 | 11.21 |  |
|  | Creatinine (umol/L) | 55 | 52 | 49 |  | 71 | 50 | 54 |  | 52 | 48 | 11.46 | 40 |  |
|  | LDH（U/L） | 397.2 | 254 | 248.3 |  | 398 | 224 | 196.4 |  | 128.7 | 201.6 | 198.4 | 138 |  |
|  |  |  |  |  |  |  |  |  |  |  |  |  |  |  |
| **Patient 8** | Lymphocyte count (X10^9^/L) | 0.61 | 0.07 | 0.12 | 0.27 | 0.41 | 0.18 | 0 |  |  | 0.01 | 0.1 | 0.12 | 0.45 |
|  | Leukocyte count (X10^9^/L) | 0.69 | 0.16 | 0.21 | 0.4 | 0.49 | 0.21 | 0.04 | 0.24 | 0.04 | 0.02 | 3.09 | 3.04 | 4.61 |
|  | Hemoglobin (g/L) | 76 | 70 | 73 | 63 | 74 | 66 | 74 | 58 | 64 | 72 | 68 | 81 | 90 |
|  | Platelet (X10^9^/L) | 69 | 32 | 9 | 45 | 23 | 11 | 4 | 81 | 25 | 13 | 22 | 15 | 31 |
|  |  |  |  |  |  |  |  |  |  |  |  |  |  |  |
|  | ALT（U/L） | 17.1 |  | 32.8 | 26.6 | 46.9 | 39.5 | 90.2 | 26.8 | 19.2 | 11.2 | 13.9 | 13.7 | 50.8 |
|  | AST（U/L） | 20.9 |  | 19 | 11.7 | 29.9 | 34.6 | 58.8 | 22.3 | 14.2 | 11.7 | 14.3 | 19.3 | 38.3 |
|  | Total bilirubin (umol/L) | 7.8 |  | 9.06 | 4.56 | 6.24 | 4.43 | 11 | 15.3 | 11.94 | 12.4 | 8.6 | 11.07 | 18 |
|  | Direct bilirubin (umol/L) | 2.72 |  | 3.39 | 2.31 | 2.7 | 1.88 | 4.47 | 5.47 | 5.61 | 5.88 | 5.1 | 6.14 | 7.13 |
|  | Creatinine (umol/L) | 48 | 41 | 40 | 38 | 44 | 47 | 35 | 31 | 39 | 36 | 40 | 52 | 38 |
|  | LDH（U/L） | 254 |  | 231 | 239 | 256 | 259 | 288 | 352 | 277 | 272 | 188 | 256 | 393 |
|  |  |  |  |  |  |  |  |  |  |  |  |  |  |  |
| **Patient 9** | Lymphocyte count (X10^9^/L) | 0.03 | 0.01 | 0.1 | 0.17 | 0.52 |  | 0 | 0 |  | 0.15 | 2.41 |  |  |
|  | Leukocyte count (X10^9^/L) | 1.94 | 0.17 | 0.13 | 0.2 | 0.54 |  | 0.01 | 0.01 |  | 0.85 | 3.82 |  |  |
|  | Hemoglobin (g/L) | 71 | 82 | 69 | 58 | 88 |  | 60 | 73 |  | 68 | 142 |  |  |
|  | Platelet (X10^9^/L) | 23 | 37 | 11 | 29 | 12 |  | 17 | 4 |  | 32 | 136 |  |  |
|  |  |  |  |  |  |  |  |  |  |  |  |  |  |  |
|  | ALT（U/L） | 10.4 | 8.5 | 7.4 | 23.9 |  |  | 168.6 | 105.5 |  | 51.6 | 122.1 |  |  |
|  | AST（U/L） | 15.8 | 11.6 | 10.3 | 25.3 |  |  | 79.6 | 60.2 |  | 23.7 | 28.8 |  |  |
|  | Total bilirubin (umol/L) | 13.9 | 11.96 | 8.95 | 11.83 |  |  | 10.45 | 11.3 |  | 10.45 | 11.3 |  |  |
|  | Direct bilirubin (umol/L) | 5.03 | 5.02 | 4.3 | 4.67 |  |  | 6.13 | 6.4 |  | 3.99 | 4.09 |  |  |
|  | Creatinine (umol/L) | 43 |  | 31 | 30 | 33 |  | 37 |  |  |  | 30 |  |  |
|  | LDH（U/L） | 303 |  |  | 141.3 | 236.1 |  | 163.3 |  |  | 177.7 | 533.4 |  |  |
|  |  |  |  |  |  |  |  |  |  |  |  |  |  |  |
| **Patient 10** | Lymphocyte count (X10^9^/L) | 0.04 |  | 0.24 | 0.13 | 0.25 | 0.55 |  |  |  |  |  |  |  |
|  | Leukocyte count (X10^9^/L) | 0.36 | 0.02 | 0.31 | 0.19 | 0.31 | 0.6 |  |  |  |  |  |  |  |
|  | Hemoglobin (g/L) | 112 | 128 | 97 | 90 | 90 | 81 |  |  |  |  |  |  |  |
|  | Platelet (X10^9^/L) | 19 | 15 | 24 | 35 | 34 | 23 |  |  |  |  |  |  |  |
|  |  |  |  |  |  |  |  |  |  |  |  |  |  |  |
|  | ALT（U/L） | 31.8 |  | 47.4 | 31.7 |  | 28.7 |  |  |  |  |  |  |  |
|  | AST（U/L） | 18 |  | 27.4 | 44.9 |  | 48.3 |  |  |  |  |  |  |  |
|  | Total bilirubin (umol/L) | 5.8 |  | 8.9 | 7.3 |  | 10.4 |  |  |  |  |  |  |  |
|  | Direct bilirubin (umol/L) | 2.42 |  | 4.31 | 3.65 |  | 4.22 |  |  |  |  |  |  |  |
|  | Creatinine (umol/L) | 81 |  | 70 | 69 |  | 67 |  |  |  |  |  |  |  |
|  | LDH（U/L） | 136.8 |  | 152.4 | 303 |  | 337.3 |  |  |  |  |  |  |  |

ALT: Alanine transaminase; AST: aspartate aminotransferase; LDH: Lactate dehydrogenase.

1 Philip, B. *et al.* A highly compact epitope-based marker/suicide gene for easier and safer T-cell therapy. *Blood* **124**, 1277-1287, doi:10.1182/blood-2014-01-545020 (2014).

2 Chai, X., Jin, X. & Zhao, M. F. [Development and functional verification of CAR-T cells targeting CLL-1]. *Zhonghua Xue Ye Xue Za Zhi* **43**, 102-106, doi:10.3760/cma.j.issn.0253-2727.2022.02.003 (2022).

3 Lee, D. W. *et al.* ASTCT Consensus Grading for Cytokine Release Syndrome and Neurologic Toxicity Associated with Immune Effector Cells. *Biol. Blood Marrow Transplant.* **25**, 625-638, doi:10.1016/j.bbmt.2018.12.758 (2019).
